# Subxiphoid Single-Port Robotic Thymectomy Using the Single-Port Robotic System versus VATS: A Multi-Institutional, Retrospective, and Propensity Score-Matched Study

**DOI:** 10.3390/cancers16162856

**Published:** 2024-08-15

**Authors:** Jun Hee Lee, Jinwook Hwang, Tae Hyun Park, Byung Mo Gu, Younggi Jung, Eunjue Yi, Sungho Lee, Soon Young Hwang, Jae ho Chung, Hyun Koo Kim

**Affiliations:** 1 Department of Thoracic and Cardiovascular Surgery, Guro Hospital, Korea University College of Medicine, Seoul 02841, Republic of Korea; lee2632@naver.com (J.H.L.); lyukhson9@gmail.com (T.H.P.); luvotomy7@naver.com (B.M.G.); 2 Department of Thoracic and Cardiovascular Surgery, Ansan Hospital, Korea University College of Medicine, Ansan 15355, Republic of Korea; znuke75@korea.ac.kr; 3 Department of Thoracic and Cardiovascular Surgery, Anam Hospital, Korea University College of Medicine, Seoul 02841, Republic of Korea; schifer@naver.com (Y.J.); viking99@hanmail.net (E.Y.); sholeemd@gmail.com (S.L.); 4 Department of Biostatistics, Korea University College of Medicine, Seoul 02841, Republic of Korea; hwangsy214@gmail.com

**Keywords:** robotics, robotic-assisted thoracic surgery, single-port, subxiphoid, thymectomy, video-assisted thoracic surgery

## Abstract

**Simple Summary:**

Subxiphoid single-port robotic-assisted thoracic surgery thymectomy using the single-port robotic system is currently being developed for minimally invasive surgery. This retrospective multi-institutional study is the largest study of its kind on this technique and is the first to compare the perioperative outcomes of this technique to those of subxiphoid single-port video-assisted thoracic surgery thymectomy. This novel technique was associated with a lower conversion rate to multi-port surgery, shorter chest tube drainage duration, and shorter postoperative hospital stays, demonstrating its potential as a surgical treatment option for selected patients.

**Abstract:**

Subxiphoid thymectomy is a novel alternative to the transthoracic approach and sternotomy, with potential benefits, such as reduced postoperative pain and faster recovery. We previously reported the initial experience with subxiphoid single-port robotic-assisted thoracic surgery (SRATS) thymectomy using the single-port robotic system (SPS). However, the efficacy of this technique remains unknown. Thus, this study examined the multi-institutional experience with SRATS thymectomy and compared the perioperative outcomes of this technique to those of subxiphoid single-port video-assisted thoracic surgery (SVATS) thymectomy. The data of patients who underwent subxiphoid SRATS and SVATS thymectomy, performed by three thoracic surgeons at three institutions between September 2018 and May 2024, were retrospectively collected. In total, 110 patients were included, with 85 and 25 undergoing SRATS and SVATS thymectomy, respectively. After propensity score matching, 25 patients were included in each group. The SRATS group was associated with a lower conversion rate to multi-port surgery (0% vs. 20%, *p* = 0.05), shorter chest tube drainage duration (1.32 ± 0.75 vs. 2.00 ± 1.29 days, *p* = 0.003), and a shorter postoperative hospital stay (2.52 ± 1.00 vs. 5.08 ± 5.20 days, *p* = 0.003). Subxiphoid SRATS thymectomy using the SPS is feasible and is a good alternative to conventional thymectomy. Further studies are necessary to confirm its benefits.

## 1. Introduction

Thymic epithelial tumors are the most prevalent type of anterior mediastinal masses in adults and are a rare form of thoracic tumor, with an incidence rate of 0.23–0.30 per 100,000 [1,2]. Thymectomy is the standard treatment for these tumors [1,3]. For decades, median sternotomy has been the gold-standard approach for thymectomy, providing excellent exposure of the thymic tissue; however, it is associated with high postoperative morbidity. Minimally invasive thymectomy, such as thymectomy via video-assisted thoracic surgery (VATS) and robotic-assisted thoracic surgery (RATS), has recently become a popular alternative procedure. Three recent meta-analyses have shown that minimally invasive thymectomy achieves better cosmetic outcomes, lower postoperative complication rates, lower blood loss, and shorter hospital stays, with comparable oncologic outcomes to those of open thymectomy [4,5,6].

Robotic surgery provides many advantages, including improved three-dimensional imaging, hand–eye coordination, freedom of movement, and tremor filtration [7]. Therefore, RATS thymectomy may enable the meticulous dissection and safe handling of thymic tissue and vessels [8,9]. Since its first report in 2001 [10], RATS thymectomy has gradually increased in popularity, with the lateral transthoracic approach initially serving as the standard approach for this technique.

Subxiphoid RATS thymectomy is emerging as an alternative approach with several advantages, including easier specimen removal, reduced postoperative pain, and a better surgical view of the upper thymic pole and bilateral phrenic nerves [11,12]. The procedure was initially performed using a subxiphoid port with two bilateral transthoracic ports [13]. However, some surgeons have endeavored to reduce the number of ports in subxiphoid RATS thymectomy to achieve a more minimally invasive surgery. We also initially attempted two-port subxiphoid RATS thymectomy using the da Vinci Xi robotic surgical system (Xi) (Intuitive Surgical Inc., Sunnyvale, CA, USA) with a subxiphoid port and an additional unilateral transthoracic port, depending on the tumor location [14]. As robotic technology developed, we attempted to perform a subxiphoid single-port RATS (SRATS) thymectomy using the da Vinci Single-Site^TM^ platform (DVSSP) (Intuitive Surgical Inc.) [15]. However, DVSSP has several limitations, including the lack of articulating instruments and collisions between instruments.

The da Vinci SP robotic surgical system (SPS) (Intuitive Surgical Inc.) has recently been developed for single-port (SP) surgery. This system was first approved for general thoracic surgery in South Korea by the Korean Food and Drug Administration in 2020. The use of the SPS in general thoracic surgery may provide improved cosmetic results, reduced postoperative pain, and early recovery. Our previous reports have demonstrated its feasibility in general thoracic surgery, including mediastinal mass excision and thymectomy [16,17]. We have also gained a decade of experience in performing subxiphoid single-port VATS (SVATS) thymectomy. Although we believe that subxiphoid single-port RATS (SRATS) thymectomy has potential benefits over subxiphoid SVATS thymectomy, no study has compared the two procedures. Therefore, this study examined multi-institutional experience with subxiphoid SRATS thymectomy using the SPS and compared the perioperative outcomes of this technique to those of subxiphoid SVATS thymectomy.

## 2. Materials and Methods

### 2.1. Study Design and Patient Selection

We examined the data of patients who underwent subxiphoid SRATS and SVATS thymectomy performed by three thoracic surgeons (JHC, JWH, and HKK) at three institutions in South Korea between September 2018 and March 2024. All three surgeons in our study performed RATS and VATS for more than 100 cases. All patients in the SRATS group underwent subxiphoid SRATS thymectomy using the SPS, and all patients in the SVATS group underwent subxiphoid SVATS thymectomy. The procedure selection depended on tumor characteristics, patient preference, and surgeon preference.

The indications and contraindications for both procedures were similar to those applied with conventional minimally invasive thymectomy. Height and obesity were not considered contraindications. However, creating a subxiphoid tunnel in patients with obesity may require additional time, potentially prolonging the total operative time. Furthermore, from an anatomical perspective, an elongated xiphoid process, inwardly curved xiphoid process, or pectus excavatum may constitute challenges for the subxiphoid approach. Tumor invasion of the great vessels was an absolute contraindication for SRATS thymectomy. Relative contraindications included a history of radiotherapy, tumor size of >8 cm, and tumors invading other organs or structures [12,15,17].

Data on preoperative clinical variables, including age, sex, comorbidities, American Society of Anesthesiologists score, body mass index, neurological examination, blood tests, and chest computed tomography (CT), were collected. Chest CT was routinely performed for all patients. If the chest CT indicated a high possibility of thymic malignancy, thymectomy was recommended; CT-guided percutaneous biopsy was not usually performed. When a differential diagnosis between thymic malignancy and thymic cyst or hyperplasia was necessary on chest CT imaging, chest magnetic resonance imaging (MRI) was conducted. If the MRI indicated a high possibility of thymic malignancy, thymectomy was recommended. Additionally, chest MRI and PET CT were conducted to identify anatomical structures and assess surgical feasibility. Preoperative neurological assessments with the diagnosis of myasthenia gravis (MG) were conducted by a neurologist.

Intraoperative outcomes (including total operative time, docking time, estimated blood loss, and any intraoperative complications) and postoperative outcomes (such as postoperative pain, chest tube duration, postoperative hospital stay, pathological data, and postoperative complications) were recorded. Extended thymectomy was defined as the complete resection of the whole thymus with the surrounding adipose tissue. The pathological results were categorized based on the World Health Organization classification with the tumor–node–metastasis staging system. Postoperative complications were classified using the Clavien–Dindo classification [18].

### 2.2. Creation of the Subxiphoid Tunnel

**Figure 1:**
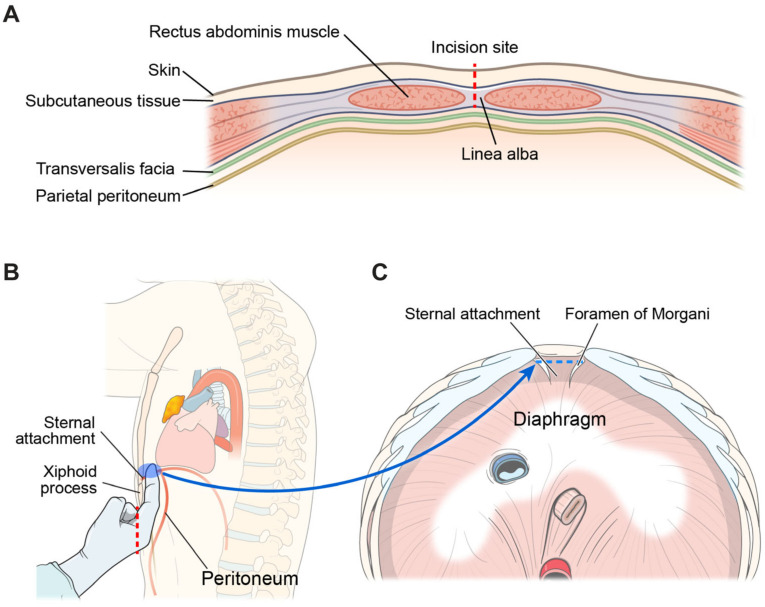
Anatomy for the creation of the subxiphoid tunnel. (**A**) The red dotted line indicates the incision line. Notably, the peritoneum should be preserved. (**B**) Blunt dissection of the retrosternal space using a finger. The sternal attachment of the diaphragm (blue box) can be safely dissected without diaphragmatic injury. (**C**) The blue dotted line indicates the incision line in the sternal attachment of the diaphragm.

The patient was placed in the open-legged supine position under general anesthesia. Two-lung ventilation with a low tidal volume was the routine ventilation strategy, whereas one-lung ventilation was used for patients with tumors invading adjacent structures. A vertical incision measuring 2.5–4 cm was made 1–2 cm below the xiphoid process without its removal. The skin and subcutaneous fat and linea alba were carefully dissected to avoid damaging the peritoneum (Figure 1A). The preperitoneal space and sternal attachment of the diaphragm were bluntly dissected using a finger (Figure 1B,C). A multi-channel port, such as the Lapsingle Vision (Sejong Medical, Gyeonggi, Republic of Korea) or SP Access Port (Intuitive Surgical Inc.), was installed through a subxiphoid incision. Carbon dioxide (CO_2_) gas was insufflated through a multi-channel port at a pressure of 6–10 mmHg.

### 2.3. Surgical Technique: Subxiphoid SRATS Thymectomy

**Figure 2:**
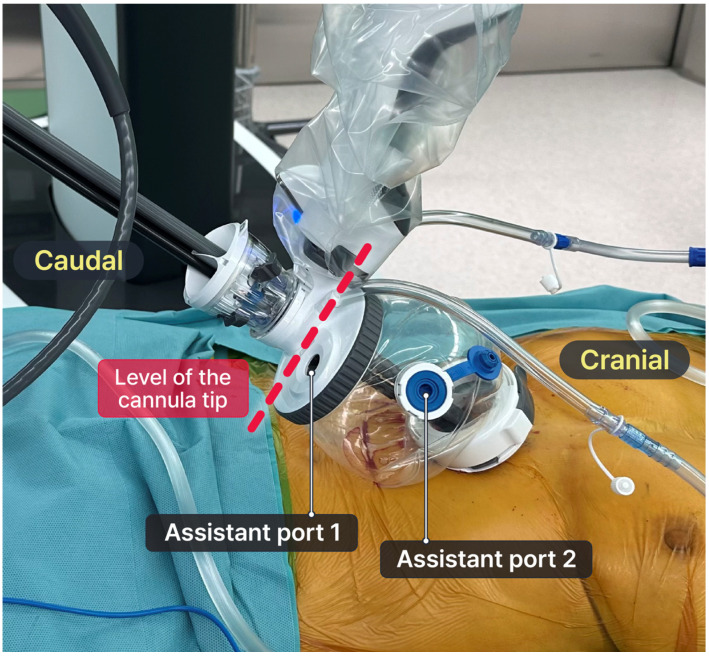
The floating dock technique with an SP access port (Intuitive Surgical Inc.). The red dotted line indicates the level of the cannula tip. The SP access port has two assistant ports (white lines).

In the early stages, the pericardial fat and tissue in the lower mediastinal area were dissected using VATS due to the technical limitations of the SPS, requiring a minimum distance of 10 cm between the cannula and the target lesion. The details of the RATS–VATS hybrid approach were provided in our previous study [15,16]. However, in this study, we performed pure robotic surgery after utilizing the SP Access Port with the floating dock technique [17]. The floating dock technique was necessary for pure robotic surgery in shallow spaces, specifically opening the mediastinum during thymectomy. The cannula tip was docked onto the tip of the multi-channel port and floated above the incision [17,19]. The port of entry floats above the incision, enabling the achievement of sufficient space from the cannula tip to the target lesion, thereby allowing for the best range of motion within a confined space (Figure 2). The bedside assistant can insert their instrument, including a suction device and an endoscopic stapler Signia™ stapling system (Medtronic, Minneapolis, MN, USA), through the assistant port in the SP access port without the need for an additional port.

**Figure 3:**
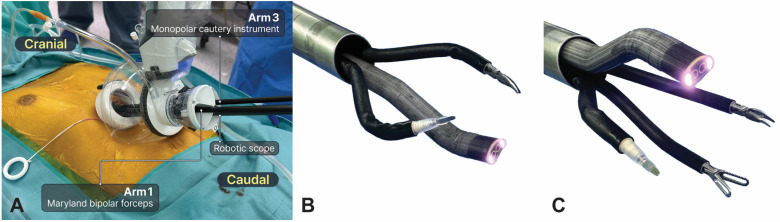
Subxiphoid single-port robotic-assisted thoracic surgery thymectomy using the da Vinci single-port robotic surgical system. (**A**) Floating dock technique using the SP access port. A robotic scope was inserted at the 6 o’clock position to obtain the above view. (**B**) Initial phase: the robotic scope was positioned in the lower middle hole (6 o’clock position). Dissection was commenced using Maryland bipolar forceps and a monopolar cautery instrument at the left (arm 1) and right (arm 2) holes, respectively. (**C**) Intermediate phase: the robot scope was repositioned in the lower middle hole (12 o’clock position) with Maryland bipolar forceps, Cadiere forceps, and a monopolar cautery instrument at arms 1, 2, and 3, respectively. This system, including three flexible arms and one flexible robotic scope, allows for single-port surgery in a confined space.

After positioning the da Vinci SP patient cart on the right side, a robotic scope was introduced into the lower middle hole (arm 2) to obtain the above view (Figure 3A). Dissection was usually performed using Cadiere forceps, Maryland bipolar forceps, and monopolar cautery instruments. The procedure initially involved the use of two arms, with Maryland bipolar forceps and a monopolar cautery instrument placed at the left and right holes, respectively (Figure 3B). The bilateral mediastinal pleura was opened after the connective tissue beneath the xiphoid process was dissected. Next, the robotic scope was repositioned at the upper middle hole to achieve the below view, whereas the Cadiere forceps were added at the lower middle hole (arm 2) (Figure 3C). The surgical procedure is similar to that of the conventional subxiphoid RATS thymectomy. The dissection of thymic tissues was routinely performed in the caudal-to-cranial direction. The adipose tissue at the bilateral cardiophrenic angles was completely removed. After identifying the phrenic nerve as the first landmark for the lateral margin of dissection, the entire thymus gland was dissected cranially until the innominate vein was identified as the second landmark. Care must be taken to avoid damage to the phrenic nerve and innominate vein. Several thymic vessels were ligated using the robotic Hem-o-Lok. Transferring the tumor to the contralateral pleural cavity can improve the exposure of the remaining thymus, particularly for large tumors. The upper pole of the thymus was carefully dissected, and the right upper pole was pulled downward and dissected freely, followed by the dissection of the left upper pole. After the upper poles of the thymus and surrounding fatty tissue were removed, the resected specimen was extracted through the same incision using a retrieval bag. Finally, two chest drains (Evacuator Barovac, 100 mL, Sewoon Medical Co., Seoul, Republic of Korea) were inserted into the bilateral pleural cavity through a subxiphoid incision once sufficient hemostasis was achieved. A video of the surgical procedure is provided (Video S1).

### 2.4. Surgical Technique: Subxiphoid SVATS Thymectomy

**Figure 4:**
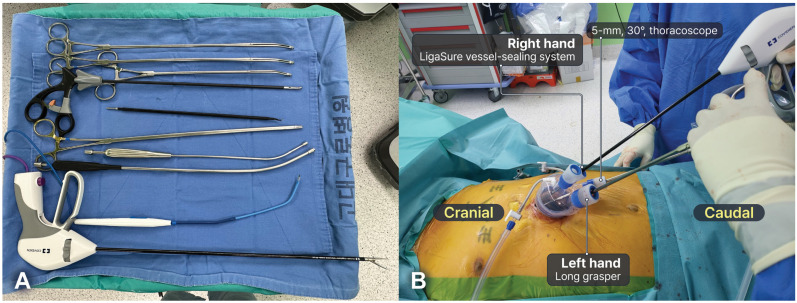
Subxiphoid single-port video-assisted thoracic surgery thymectomy. (**A**) Instruments for subxiphoid single-port video-assisted thoracic surgery thymectomy. (**B**) A total of four devices can be inserted through a multi-channel port. Surgeons commonly use a long curved grasper in their left hand and a long bipolar energy device in their right hand. However, in some cases, the surgeon can cross the hands.

The surgeon stood between the patient’s legs or on their right side, depending on their preference, whereas the assistants stood on the left side. A multi-channel port (Lapsingle Vision) was commonly used, and a 5-mm, 30° scope was typically used to minimize collisions between the scope and instruments. The Lapsingle Vision has four channels: one for the thoracoscope, two for the surgeon’s instruments, and one for the assistant’s instruments. Conventional thoracoscopic instruments designed for SVATS were also used, and long curved thoracoscopic instruments further reduced collisions between instruments (Figure 4A). A 5-mm camera scope was inserted through the most inferior part of the multi-channel port entrance, with the thoracoscopic instruments subsequently introduced through another multi-channel port entrance. Surgeons used thoracoscopic grasping forceps and a long bipolar energy device (44 cm, LigaSure, Covidien, Boulder, CO, USA) in their left and right hands, respectively (Figure 4B). A bipolar energy device was used for dissection, exposure, sealing, and cutting. Bipolar energy devices cause less thermal injury to the surrounding tissues and nerves, enabling safe dissection along the phrenic nerve and vessels [20]. The position and type of the instrument can be adjusted as needed. In some cases, crossing hands to reduce interference between instruments may be necessary. The steps performed in SVATS were similar to those in SRATS.

### 2.5. Conversion to Sternotomy or Multi-Port Surgery

In emergency situations, such as intraoperative bleeding, conversion to sternotomy is needed. Preoperative skin preparation and draping, including that of the sternum, are essential for rapid conversion to median sternotomy. A vertical incision for the subxiphoid tunnel facilitates conversion to sternotomy.

Stapling can be performed using an endoscopic stapler through an assistant port in the SP access port. If stapling is difficult or the surgical view is insufficient, an additional port is created. An additional 12-mm port can be placed in the 5th–7th intercostal space in the midclavicular line according to the target lesion if necessary.

### 2.6. Postoperative Management

The hospitals involved in this study are affiliated with the Korea University Medical Center and share similar protocols for patients with thymoma and thymic carcinoma. In both SRATS and SVATS groups, the postoperative protocols were the same. Unless contraindicated, all patients received intravenous patient-controlled analgesia, along with oral analgesics, including acetaminophen/tramadol combination tablets and nonsteroidal anti-inflammatory drugs (Ansan Hospital, Guro Hospital) or acetaminophen/tramadol/codeine combination tablets (Anam Hospital). Pain scores were assessed daily every 8 h during hospitalization using the visual analog scale (range: 0–10). Drains were removed if the drain output was less than three times the patient’s body weight (Ansan Hospital, Guro Hospital) or less than 100 mL (Anam Hospital) and if no pleural effusion was observed on lateral decubitus chest radiography. The patients were discharged the day after drain removal. Follow-up protocols were also the same (Table S1).

### 2.7. Statistical Analysis

All statistical analyses were performed using IBM SPSS Statistics for Windows (version 27 (IBM Corp., Armonk, NY, USA)) and SAS 9.4 (SAS Institute, Cary, NC, USA). Categorical variables were expressed as counts (percentages) and compared using the chi-square or Fisher’s exact tests. The Kolmogorov–Smirnov test and Shapiro–Wilk test were used to assess the distribution for continuous variables. Continuous variables were presented as mean ± standard deviation and compared using the Student’s *t*-test or Mann–Whitney U test. A two-sided *p*-value less than or equal to 0.05 was considered statistically significant.

To minimize selection bias, propensity score matching (PSM) was conducted based on potentially confounding variables, including age, sex, and pathological diagnosis. Patients were matched using a 1:1 nearest neighbor matching algorithm.

### 2.8. Ethical Statement

This study was approved by the Institutional Review Board of the three institutions involved: Korea University Anam Hospital (2024AN0298), Korea University Ansan Hospital (2024AS0154), and Korea University Guro Hospital (2024GR0241). The requirement to obtain informed consent was waived owing to the retrospective study design and given its minimal-risk nature, in accordance with institutional guidelines.

## 3. Results

This study included 110 patients, of which 85 and 25 patients underwent SRATS and SVATS, respectively (Table S2). After PSM, 25 patients were included in each group.

Table 1 summarizes patient characteristics in the two groups. Following PSM, the mean mass size was 3.68 ± 1.87 and 3.84 ± 2.01 cm in the SRATS and SVATS groups, respectively (*p* = 0.773). No significant between-group differences were found in patient characteristics (*p* > 0.05).

Table 2 shows the intraoperative outcomes of the two groups. All patients underwent successful R0 resection. There was no significant difference in the mean total operative time (154.46 ± 74.06 vs. 146.76 ± 67.07 min, *p* = 0.674). The resection of adjacent structures was performed in four (4%) patients in the SRATS group and four (16%) patients in the SVATS group. In the SRATS group, two patients required innominate vein resection using an endoscopic stapler, with one requiring conversion to multi-port surgery (requiring an additional port). Additionally, one patient underwent pericardial resection and reconstruction without conversion (Video S2). Two patients in the SVATS group required resection of the invaded lung using an endoscopic stapler, and one of them required conversion to multi-port surgery. One patient underwent phrenic nerve resection and innominate vein repair with conversion to multi-port surgery. One patient underwent pericardial resection and reconstruction with conversion to median sternotomy. No cases of conversion to sternotomy occurred in the SRATS group. However, one patient required conversion to sternotomy in the SVATS group. Notably, the conversion rate to multi-port surgery was significantly lower in the SRATS group (2% vs. 20%, *p* = 0.006). Even after PSM, the conversion rate to multi-port surgery remained significantly lower in the SRATS group (0% vs. 20%, *p* = 0.05). Table S3 summarizes the details of the patients who required conversion to sternotomy or multi-port surgery.

Table 3 summarizes the postoperative outcomes and pathology results of the two groups. Notably, the chest tube drainage duration was significantly shorter in the SRATS group than in the SVATS group (1.40 ± 0.94 vs. 2.00 ± 1.29 days, *p* = 0.001). Additionally, the postoperative hospital stay was significantly shorter in the SRATS group than in the SVATS group (2.87 ± 1.26 vs. 5.08 ± 5.20 days, *p* = 0.007). After PSM, the SRATS group was associated with shorter chest tube drainage duration (1.32 ± 0.75 vs. 2.00 ± 1.29 days, *p* = 0.003) and a shorter postoperative hospital stay (2.52 ± 1.00 vs. 5.08 ± 5.20 days, *p* = 0.003).

**Figure 5:**
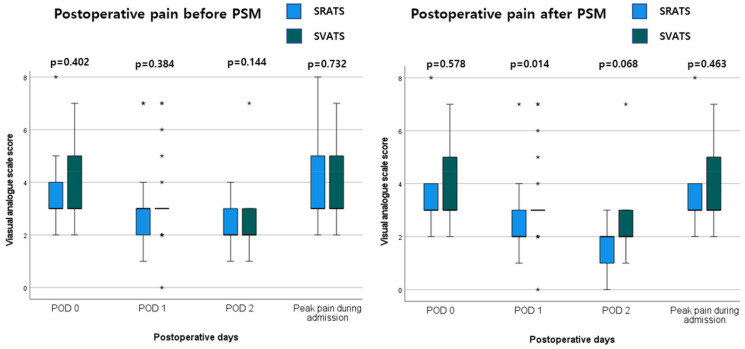
Postoperative pain on postoperative days 0, 1, and 2, as well as peak pain during admission, according to the visual analog scale score. POD: postoperative days and PSM: propensity score matching.

The postoperative pain results are presented in Figure 5. No significant differences were found in the Visual Analog Scale (VAS) score on postoperative days (POD) 0–2 and peak pain score during admission between groups (*p* > 0.05). After PSM, the SRATS group was associated with a significantly lower VAS score on POD 1 (2.60 ± 1.41 vs. 3.28 ± 1.54, *p* = 0.014).

## 4. Discussion

We investigated the experience of three Korean institutions of subxiphoid SRATS thymectomy using the SPS and compared this technique to subxiphoid SVATS thymectomy. Subxiphoid SRATS thymectomy using the SPS is not widely performed. This study aimed to establish the feasibility and benefits of subxiphoid SRATS thymectomy using the SPS. To the best of our knowledge, this study is the first to compare the perioperative outcomes of subxiphoid SRATS using the SPS to those of subxiphoid SVATS thymectomy and is the largest study to report on this novel technique. We demonstrated favorable outcomes in terms of the conversion rate to multi-port surgery, chest tube drainage duration, and postoperative hospital stay. No significant difference in the operative time between the SRATS and SVATS groups was found, although the total operative time was generally longer because an extended thymectomy was performed in almost all patients.

The SRATS group had a significantly lower conversion rate to multi-port surgery than the SVATS group, and there were no cases of conversion to median sternotomy within the SRATS group. Previous studies have reported that minimally invasive thymectomy had a lower conversion rate to median sternotomy, and our conversion rate was comparable to that reported in the literature (range: 0–14%) [9,21,22,23,24,25]. A potential reason for these positive outcomes is the improved accuracy of the procedure. Specifically, the enhanced 3-D surgical view of the robotic system using the articulating robot scope and the precise movements of the robotic instruments are useful for the upper thymic pole dissection or identification of the phrenic nerve.

Our study also demonstrated that subxiphoid SRATS thymectomy using the SPS is associated with shorter chest tube drainage duration and postoperative hospital stay. There are several possible reasons for these findings. First, the articulating robotic arm and robot scope provide an improved surgical view, allowing meticulous dissection. This facilitates delicate and complex procedures, allowing the complete removal of thymic tissue and the surrounding adipose tissue with better hemostasis and reduced damage to surrounding tissues. Therefore, chest drainage is reduced with shorter chest tube duration and postoperative hospital stay. Second, there may have been differences in the management protocols for these procedures as SRATS was introduced in the participating institutions in November 2020, while SVATS was introduced in September 2018.

Our study showed that the SRATS group was associated with a significantly lower VAS score on POD 1 after PSM. Before PSM, there was no statistically significant difference in postoperative pain, and a significant difference was found only on POD 1 after PSM. Therefore, it is difficult to conclude that the SRATS approach results in less postoperative pain based on this result alone. Furthermore, three recent randomized controlled studies comparing VATS and RATS showed no significant difference in postoperative pain between the two approaches [26,27,28]. Gsous et al. reported that there was no significant difference in chronic pain between both approaches [29]. Further research is needed to compare postoperative pain.

The optimal approach for thymectomy remains controversial. The lateral transthoracic approach, such as the right, left, or bilateral approach, is the most commonly used approach for minimally invasive thymectomy. However, the lateral transthoracic approach has some limitations, such as the risk of intercostal nerve damage and difficulty in identifying the phrenic nerve and upper thymic pole. The unilateral transthoracic approach may not provide sufficient exposure of the contralateral phrenic nerve and pleural cavity. Therefore, the bilateral transthoracic approach should be considered for patients with MG to ensure the complete removal of all thymic tissues. However, this approach may result in increased total operative time, postoperative pain, and number of ports, as well as poor cosmetic outcomes [30].

In contrast, the subxiphoid approach for thymectomy provides an improved view of the bilateral phrenic nerves and the upper thymic pole above the innominate vein while minimizing postoperative pain. Chronic pain may be prevented using a subxiphoid incision because only cutaneous branches of intercostal nerves are present in this area. The subxiphoid approach can be applied in various fields of thoracic surgery, such as bullectomy, lung biopsy, bilateral metastasectomy, and major pulmonary resection [31,32,33,34]. Recently, three meta-analyses showed that subxiphoid VATS thymectomy is associated with less blood loss and postoperative pain, shorter chest tube duration, and shorter hospital stays than transthoracic VATS thymectomy [35,36,37]. The subxiphoid approach for thymectomy was initially attempted using a subxiphoid port with two transthoracic or two subcostal ports. In particular, the combination of a subxiphoid port with two transthoracic ports may cause intercostal nerve damage due to port placement in the intercostal space. Although combining subxiphoid and subcostal ports can minimize intercostal nerve damage, it may be technically challenging when making subcostal ports.

SP surgery in the field of general thoracic surgery has potential benefits, including reduced postoperative pain, fewer postoperative complications, faster recovery, and improved cosmetic outcomes [38,39,40]. Many surgeons who prefer SVATS have attempted subxiphoid SVATS thymectomy since this technique was first reported in 2012 [41]. Subxiphoid SVATS thymectomy is feasible but technically demanding, particularly for dissecting the upper thymic pole and cardiac-diaphragmatic angle. Inexperienced surgeons may experience poor surgical manipulability in SVATS due to collisions between thoracoscopic instruments and the scope. Therefore, surgeons must maintain an unnatural posture while standing beside the operating table. These limitations worsen surgeon ergonomics during the procedure.

Compared to subxiphoid SVATS thymectomy, subxiphoid SRATS thymectomy using the SPS improved surgeon ergonomics, surgical manipulability, and surgical view, particularly for dissecting the upper thymic pole. The SPS has several new features compared to the previous robotic system, featuring an articulating robot scope and three wristed arms that can be inserted through a single incision, allowing free movement in confined spaces, whereas the DVSSP includes only two non-flexible arms [15]. The robot scope can also be adjusted to the cobra mode, enabling it to move above or below the instruments to reduce collisions and improve the surgical view. Furthermore, the features of the SPS provide several advantages over DVSSP and VATS, including more complex movements, meticulous dissection, and fewer collisions. Therefore, the SPS is more suitable for subxiphoid thymectomy. In particular, the articulating instrument of the SPS enables a more secure and comfortable dissection around the upper mediastinum, innominate vein, and upper thymic pole in patients with a tall stature or a long chest than VATS. Furthermore, within confined spaces where instrument collisions are common, the experience of the scopist, rather than solely that of the surgeon, can be crucial to ensure clear visibility and safe dissection during SVATS, thereby introducing a certain degree of dependency. However, SRATS using the SPS enables surgeons to control instruments independently, including the articulating scope, thereby minimizing collisions and reducing dependency on an assistant. Moreover, in SVATS and DVSSP, the surgeon can typically use two instruments; however, in SRATS, the surgeon can use three instruments. This advantage can particularly facilitate retraction, especially in large-sized thymomas.

The SPS has some technical limitations. First, this system does not include a dedicated robotic stapler or vessel-sealing device. Small vessels are ligated using the robotic Hem-o-Lok, which can increase the total operative time. Although large vessels can be excised using an endoscopic stapler, this can be technically challenging and potentially time-consuming. Therefore, the development of more dedicated and advanced robotic instruments for the SPS is required. Second, this system requires a minimum distance of 10 cm between the cannula and the target lesion. The floating dock technique is essential for pure robotic surgery and requires CO_2_ insufflation. Notably, CO_2_ insufflation provides an enlarged retrosternal space without a sternal retractor. Elevating the sternum may lead to injury. One-lung ventilation is not usually performed because CO_2_ insufflation displaces the lungs and pericardium downward. However, caution should be exercised when using CO_2_ insufflation in patients with compromised cardiac output because it can limit venous return and cardiac output. In such cases, reducing CO_2_ insufflation pressure or intermittent CO_2_ insufflation can be safely performed. Third, the SPS did not include a suction system, which can make it difficult to maintain a clear view during unexpected bleeding. To address this, we place gauze in the operative field in advance, so that it could be used immediately if necessary.

Contraindications for subxiphoid SRATS thymectomy using the SPS are unknown. Previously, contraindications for robotic thymectomy included large tumors (>8 cm) and tumors that invaded the pericardium or great vessels. However, some experienced robotic surgeons have attempted robotic thymectomy in complex cases, such as resecting and reconstructing the pericardium or vessels. Pericardial reconstruction using a Gore-Tex membrane can be safely performed using the SPS. Large tumors and those invading the lungs and innominate vein are not absolute contraindications for subxiphoid SRATS thymectomy using the SPS. However, tumor invasion of the great vessels is an absolute contraindication for SRATS thymectomy using the SPS. Nonetheless, advances in robotic technology may enable more complex thymectomy procedures in the future.

To date, the SPS has not been widely used in general thoracic surgery, and there is a paucity of clinical data on subxiphoid SRATS thymectomy using the SPS [16,17]. However, we believe that the true value of the SPS lies in its potential to be ideal for thymectomy in the field of thoracic surgery. Additionally, we believe that subxiphoid SRATS thymectomy using the SPS provides potential benefits by combining the advantages of robotic surgery, SP surgery, and the subxiphoid approach. The application of a new technique always involves challenges, and our study may contribute to the widespread adoption of the SPS in the field of general thoracic surgery. With advances in SPS technologies, including the dedicated robotic stapler and vessel-sealing device, we anticipate that this system will gain popularity for thymectomy.

This study has some limitations. First, this was a non-randomized retrospective study with a small sample size. Some important data may be missing and inaccurate. Although PSM could reduce selection bias and confounders, the nature of the retrospective study could cause bias and possible confounding factors, including surgeon experience and disease severity. We could not adjust confounding factors owing to the limitations of the study design and the small sample size. Second, our study only included short-term outcomes because SRATS and SVATS were performed recently in many cases for both groups; long-term follow-up data, including oncological results, are needed owing to the indolent nature of thymic epithelial tumors. Third, the control group did not include those who underwent RATS thymectomy using the conventional Xi robotic system or thymectomy via a transthoracic approach. Finally, SVATS thymectomy was initiated several years before the start of SRATS thymectomy, potentially introducing some bias.

## 5. Conclusions

This study demonstrated that subxiphoid SRATS thymectomy using the SPS is associated with a lower conversion rate to multi-port surgery, shorter chest tube drainage duration, and a shorter postoperative hospital stay compared to subxiphoid SVATS thymectomy. Our findings suggest that subxiphoid SRATS thymectomy using the SPS is feasible and can be a good alternative to conventional minimally invasive thymectomy. Further large-scale randomized controlled studies are needed to confirm our findings.

## Figures and Tables

**Table 1:** Patient characteristics before and after propensity score matching.

	Before PSM			After PSM		
Variable	SRATS (n = 85)	SVATS (n = 25)	*p*-Value	SRATS (n = 25)	SVATS (n = 25)	*p*-Value
Age (years)	53.38 ± 12.98	57.6 ± 13.93	0.520	57.72 ± 13.69	57.6 ± 13.93	0.844
Sex (male), n (%)	33 (39%)	13 (52%)	0.240	13 (52%)	13 (52%)	1.000
BMI (kg/m^2^)	24.99 ± 3.90	24.79 ± 3.40	0.963	24.99 ± 2.81	24.79 ± 3.40	0.820
Comorbidities, n (%)						
Hypertension	21 (25%)	10 (40%)	0.135	7 (28%)	10 (40%)	0.551
Diabetes mellitus	12 (14%)	6 (24%)	0.236	5 (20%)	6 (24%)	1.000
COPD	5 (6%)	1 (4%)	1.000	3 (12%)	1 (4%)	0.609
Myasthenia gravis	7 (8%)	3 (12%)	0.692	3 (12%)	3 (12%)	1.000
ASA score	2.32 ± 0.71	2.36 ± 0.70	0.749	2.28 ± 0.79	2.36 ± 0.70	0.835
Mass size (cm)	3.29 ± 1.61	3.84 ± 2.01	0.216	3.68 ± 1.87	3.84 ± 2.01	0.773

ASA: American Society of Anesthesiology, BMI: body mass index, COPD: chronic obstructive pulmonary disease, PSM: propensity score matching, SRATS: single-port robotic-assisted thoracic surgery, and SVATS: single-port video-assisted thoracic surgery.

**Table 2:** Intraoperative outcomes before and after propensity score matching.

	Before PSM			After PSM		
	SRATS (n = 85)	SVATS (n = 25)	*p*-Value	SRATS (n = 25)	SVATS (n = 25)	*p*-Value
Completeness of resection (R0)	85 (100%)	25 (100%)	1.000	25 (100%)	25 (100%)	1.000
Extent of resection, n (%)			0.076			0.235
Extended thymectomy	83 (98%)	22 (88%)		25 (100%)	22 (88%)	
Partial thymectomy	2 (2%)	3 (12%)		0	3 (12%)	
Resected adjacent structures, n (%)						
Lung	1 (1%)	2 (8%)	0.129	1 (4%)	2 (8%)	1.000
Innominate vein	2 (2%)	0	1.000	0	0	
Phrenic nerve	0	1 (4%)	0.227	0	1 (4%)	1.000
Pericardium	1 (1%)	1 (4%)	0.405	1 (4%)	1 (4%)	1.000
Anesthesia time (min)	225.88 ± 79.46	214.40 ± 71.59	0.724	236.00 ± 96.41	214.40 ± 71.59	0.640
Total operative time (min)	154.46 ± 74.06	146.76 ± 67.07	0.674	159.72 ± 86.54	146.76 ± 67.07	0.641
Conversion, n (%)						
To median sternotomy	0	1 (4%)	0.227	0	1 (4%)	1.000
To multi-port surgery	2 (2%)	5 (20%)	0.006	0	5 (20%)	0.050
Transfusion, n (%)	0	1 (4%)	0.227	0	1 (4%)	1.000

PSM: propensity score matching, SRATS: single-port robotic-assisted thoracic surgery, and SVATS: single-port video-assisted thoracic surgery.

**Table 3:** Postoperative outcomes and pathology results before and after propensity score matching.

	Before PSM			After PSM		
	SRATS (n = 85)	SVATS (n = 25)	*p*-Value	SRATS (n = 25)	SVATS (n = 25)	*p*-Value
Chest tube drainage duration (days)	1.40 ± 0.94	2.00 ± 1.29	0.001	1.32 ± 0.75	2.00 ± 1.29	0.003
Postoperative hospital stays (days)	2.87 ± 1.26	5.08 ± 5.20	0.007	2.52 ± 1.00	5.08 ± 5.20	0.003
Postoperative complications (Clavien–Dindo), n (%)			0.104			0.414
None	80 (94%)	21 (84%)		24 (96%)	21 (84%)	
I	2 (2%)	2 (8%)		1 (4%)	2 (8%)	
II	1 (1%)	1 (4%)		0	1 (4%)	
IIIa	2 (2%)	0		0	0	
IIIa/IV	0	1 (4%)		0	1 (4%)	
Pathological diagnosis, n (%)			0.882			0.932
Thymoma	36 (42%)	10 (40%)		11 (44%)	10 (40%)	
Thymic carcinoma	7 (8%)	1 (4%)		1 (4%)	1 (4%)	
Benign cystic lesions	32 (38%)	10 (40%)		8 (32%)	10 (40%)	
Other	10 (12%)	4 (16%)		5 (20%)	4 (16%)	
WHO classification, n (%)			0.551			0.812
A	8 (19%)	1 (9%)		3 (25%)	1 (9%)	
AB	14 (32%)	2 (18%)		4 (33%)	2 (18%)	
B1	5 (12%)	3(27%)		2 (17%)	3 (27%)	
B2	5 (12%)	3 (27%)		1 (8%)	3 (27%)	
B3	4 (9%)	1(9%)		1 (8%)	1 (9%)	
C	7 (16%)	1(9%)		1 (8%)	1 (9%)	
T stage, n (%)			0.558			1.000
T1a	35 (81%)	10 (91%)		10 (83%)	10 (91%)	
T1b	2 (5%)	1 (9%)		1 (8%)	1 (9%)	
T2	5 (12%)	0		1 (8%)	0	
T3	1 (2%)	0		0	0	

PSM: propensity score matching, SRATS: single-port robotic-assisted thoracic surgery, SVATS: single-port video-assisted thoracic surgery, and WHO: World Health Organization.

## Data Availability

The data underlying this article will be shared upon reasonable request to the corresponding author.

## Supplementary Materials

The following supporting information can be downloaded at: https://www.mdpi.com/article/10.3390/cancers16162856/s1, Table S1: Postoperative follow-up protocols for patients with thymoma and thymic carcinoma; Table S2: Distribution of cases according to the institution; Table S3: Summary of patients who required conversion to sternotomy or multi-port surgery; Video S1: Subxiphoid single-port robotic thymectomy using the da Vinci single-port robotic surgical system; Video S2: Pericardial resection and reconstruction during subxiphoid single-port robotic thymectomy using the da Vinci single-port robotic surgical system.
