# Supplementary material for: Subxiphoid Single-Port Robotic Thymectomy Using the Single-Port Robotic System versus VATS: A Multi-Institutional, Retrospective, and Propensity Score-Matched Study
Source: Cancers (Basel). 2024 Aug 15;16(16):2856. doi: 10.3390/cancers16162856 (PMC11353098; doi:10.3390/cancers16162856)
Supplement: Supplementary file 1 [file cancers-16-02856-s001.zip › cancers-3117110-Tables.pdf]

Table S1. Postoperative Follow-up protocols for patients with thymoma and thymic carcinoma.

Follow-up schedule																							
Months	0.5	1	3	6	12	18	24	30	36	42	48	54	60	66	72	78	84	90	96	102	108	114	120
Outpatient clinic	x	x	x	x	x	x	x	x	x	x	x	x	x	x	x	x	x	x	x	x	x	x	x
Blood test	x																						
Chest x-ray	x	x	x	x	x	x	x	x	x	x	x	x	x	v	x	x	x	x	x	x	x	x	x
Chest CT contrast		x																					
Chest CT non-contrast			x	x	x	x	x	x	x	x	x	x	x	x	x	x	x	x	x	x	x	x	x
PET-CT	When metastasis or recurrence is suspected																						

CT computed tomography, ***PET-CT*** Positron emission tomography-computed tomography

Table S2. Distribution of cases according to the institution.

	Guro Hospital	Anam hospital	Ansan hospital
SRATS (n=85)	72	7	6
SVATS (n=25)	20	5	0

SRATS single-port robotic-assisted thoracic surgery, *SVATS* single-port video-assisted thoracic surgery.

Table S3. Summary of patients who required conversion to sternotomy or multi-port surgery.

Groups	No.	Conversion to	Reasons for conversion	Total operative time (min)
SRATS	1	Multi-port surgery (Two-port)	Tumor abutting left phrenic nerve	295
	2	Multi-port surgery (Three-port)	Tumor invading innominate vein (resection of the innominate vein)	400
SVATS	1	Multi-port surgery (Three-port)	for better surgical view of the upper thymic pole	200
	2	Multi-port surgery (Two-port)	for better surgical view of the upper thymic pole	230
	3	Multiport surgery (Two-port)	Tumor invading right upper lobe lung (resection of lung)	127
	4	Multiport surgery (Three-port)	Adhesion in the cervical area due to a past history of thyroidectomy	103
	5	Multiport surgery (Three-port)	Tumor abutting left phrenic nerve (resection of the phrenic verve) Innominate vein injury (primary repair)	280
	6	Median sternotomy	Tumor invading pericardium (Pericardial resection and reconstruction)	275

SRATS single-port robotic-assisted thoracic surgery, *SVATS* single-port video-assisted thoracic surgery.
